# Nutrient Profiling Is Needed to Improve the Nutritional Quality of the Foods Labelled with Health-Related Claims

**DOI:** 10.3390/nu11020287

**Published:** 2019-01-29

**Authors:** Urška Pivk Kupirovič, Krista Miklavec, Maša Hribar, Anita Kušar, Katja Žmitek, Igor Pravst

**Affiliations:** 1 Nutrition Institute, Tržaška cesta 40, SI-1000 Ljubljana, Slovenia; urska.pivk.kupirovic@nutris.org (U.P.K.); krista.miklavec@nutris.org (K.M.); masa.hribar@nutris.org (M.H.); anita.kusar@nutris.org (A.K.); katja.zmitek@vist.si (K.Ž.); 2 VIST—Higher School of Applied Sciences, Gerbičeva cesta 51a, SI-1000 Ljubljana, Slovenia

**Keywords:** food supply, health claim, nutrition claim, nutrient profile, nutrition quality, sales data, Slovenia

## Abstract

Health-related claims on food products influence consumers and their food preferences. None of the European countries have restricted the use of health claims to foods of high nutritional quality despite the regulatory background provided by the European Union in 2006. We evaluated the nutritional quality of foods labelled with claims available in the Slovenian market using two nutrient profile models—Food Standards Australia New Zealand (FSANZ) and European World Health Organization Regional office for Europe model (WHOE)—and compared the results to the nutritional quality of all available foods. Data for prepacked foods in the Slovenian food supply were collected in 2015 on a representative sample (*n* = 6619) and supplemented with 12-month product sales data for more accurate assessments of the food supply. A considerable proportion of foods labelled with any type of health-related claim was found to have poor nutritional quality. About 68% of the foods labelled with health-related claims passed FSANZ criterion (75% when considering sales data) and 33% passed the WHOE model (56% when considering sales data). Our results highlight the need for stricter regulations for the use of health-related claims and to build upon available nutrient profiling knowledge to improve nutrition quality of foods labeled with health-related claims.

## 1. Introduction

Nutrition has an important role in the prevention of noncommunicable diseases [1]. Reformulating processed foods is now considered as a major means to help reduce the prevalence of diet-related diseases [2]. Considering this, the food industry has come under increasing pressure to improve the nutritional quality of processed foods. Food reformulation initiatives are mostly aimed at reducing salt, saturated fatty acids, trans-fatty acids, sugars, and total energy. While in some environments self-regulation and voluntary codes of practice can produce positive results, this is not always the case [3]. In cases where public health objectives oppose the commercial interests of the industry, regulatory interventions might become the most effective option [4]. However, in a global food environment, a harmonized regulatory approach is needed. 

Insights into the composition of the available processed foods in the marketplace are key input information for any food supply assessment, particularly when investigating regulatory interventions [5,6]. Ongoing monitoring of changes in the food supply has the potential to drive changes in the nutrient composition of processed foods by highlighting those that are making advances and those that are not [7]. In 2010, The Global Food Monitoring Initiative established a global branded food composition database to track the nutritional content of foods and publish comparisons between countries, food companies, and over time [5,8,9]. Accurate food labelling and composition databases are a key (but underexploited) source of data to provide governments, industry nutritionists, health professionals, and advocacy groups with new evidence to drive changes in the nutrient composition of processed food necessary for population health [8]. 

Nutrition and health claims (NHC) could be a possible driver of healthy food choices [10] if such claims were to actually be used on »healthy« foods. In our study, we evaluated the use of nutrition and health claims as defined within the European Union (EU) Regulation 1924/2006 on nutrition and health claims [11]. A ’nutrition clam’ refers to any statement, suggestion, or implication that a food is beneficial due to its caloric value or nutrients and/or other substances included or removed from the product, while a health claim provides a linkage between a food, food category or a constituent in a food and health. Existing evidence shows that such claims are attractive for consumers and can effectively affect food choices [10], at least in some population groups. However, in the European Union, nutrition and/or health claims are still found on products of lower nutritional quality, which can mislead consumers into believing that products with such claims are of better nutritional quality [12,13]. Part of the regulation that has foreseen establishment of conditions for use of nutrition and health claims, including nutrient profiles, has not yet been implemented, although over a decade has passed since the EU published this Regulation on nutrition and health claims [11]. Within the framework of the European Commission’s regulatory fitness and performance (REFIT) program, the European Commission (EC) [14] is currently seeking to evaluate whether a nutrient profiling is necessary, and whether the failure to implement nutrient profiling has had any positive or negative effect. An official report on this topic, which will provide a point-of-departure for future policies in the EU, is expected in 2019. Contrary to situation in Europe is an example of Australia and New Zealand, where FSANZ standard was implemented to determine the eligibility of a F&B to carry health claims [15].

The prevalence of NHC on prepacked foods in Slovenia was first investigated in 2011 [16]; however, as labelling of the nutrition declaration was voluntary at that time, assessment of the nutritional quality of the food supply would be challenging. This has changed with the new EU Food labelling regulation No. 1169/2011, which provided mandatory nutrition information on food labels by end of 2016. A major step forward was an assessment of the nutritional quality of pre-packaged foods carrying health-related claims, which was conducted within the European Commission-funded research project “Role of health-related claims and symbols in consumer behavior (CLYMBOL)” on a sample of foods, collected in 2013. A strength of the study was that foods were collected in five different EU countries. However, the total sample, consisted of about 400 foods per country (2034 foods in total) and we did not have access to market-share data for those products [17]. A conclusion of that study was that foods carrying health-related claims have only marginally better nutrition profiles than those that do not carry such claims. Similar finding was confirmed in the study examining nutritional quality of F&B in Canada [18]. NHC claims have higher prevalence in Canada than in Europe. The study was done on a large set of samples 15184 and found out that only 58% of F&B have high enough nutritional quality to pass FSANZ nutrient profile. 

Although the overall nutritional quality of prepacked-foods in the Slovenian food supply has not been investigated before, a number of studies focused into specific nutrients. For example, an approach with market shares data has been used to investigate sodium [7] and sugar [19] content. The importance of using sales data in investigation of the food supply was also most clearly shown in a salt iodization study, where 59% of the available products were found to be iodized, but after considering sales-data, these products represented over 95% of the market share [20].

After compiling a large database of prepacked foods in Slovenia, and with access to both nutrition composition and sales data, we were in position to conduct accurate assessments of the food supply. The objective of our study was to investigate if the overall nutritional quality of foods labelled with NHC is better when compared with all available foods, and to determine if claims are also being used on foods with poor nutritional quality. Analyses were performed using two different nutrient profiling models: the Food Standards Australia New Zealand (FSANZ) Nutrient Profiling Scoring Criterion (NPSC) [15] and the WHO Regional Office for Europe (WHOE) nutrient profile model [21]. The sales-weighting approach was used to better estimate the consumer choice with the over-availability of products in stores.

## 2. Materials and Methods

### 2.1. Food Database

Cross-sectional data on the nutritional composition of prepacked foods in the Slovenian food supply were collected during January–February 2015 in Ljubljana, Slovenia. To ensure the samples’ high representativeness, we selected grocery stores of retailers with accessible nation-wide store networks and the largest market shares (Spar, Mercator, and Hofer), which included five locations (two mega markets: Mercator center Ljubljana, Interspar Vič Ljubljana; two supermarkets: Spar Vrhovci Ljubljana, Mercator Cesta na Brdo Ljubljana; and a discount market: Hofer Brdo Ljubljana). These retailers accounted for the majority of the total national market share in terms of sales value (more than 50%) and operated in all parts of the country. In agreement with the retailers, all available prepacked products with a unique European/International Article Number (EAN) barcode were systematically photographed and recorded in the online Composition and Labelling Information System (CLAS) database of the Nutrition Institute (Ljubljana, Slovenia). The database is supported by a specially developed computer application that enables digital recognition of EAN codes, which accelerated the database’s formation and enabled the avoidance of duplicate entries. Using this online application, product photographs were used to collect product information, including data on nutrition composition. Extended list of information on a total of 10,674 unique items were collected, including the product EAN barcode, name, brand name, list of ingredients, use of additives, presence of allergens, preparation needed, portion suggestion, nutritional values, packaging volume, price, and use of health claim, nutrition claim, and symbols. The content of fruits, vegetables, and nuts (needed for nutrient profilin) was estimated according to a previously described method [22]. Flour, spices, sugar, food supplements, and as all alcoholic beverages were excluded from the data acquisition. 

According to the European Union, legislation on the labelling of nutrition information was not yet mandatory at the time of sampling in Slovenia (mandatory labelling of nutrition declaration was introduced in December 2016); therefore, only items labelled with a nutrition declaration were included in this study. In cases where nutrition information was labelled, but specific information was missing (mostly for the content of dietary fiber, which is not part of mandatory food labelling in the new regulation), the missing nutritional values were supplemented from a food composition database according to a previously described method [22]. This was required for 2356 products. As some foods (341) cannot be consumed as they are sold, their nutritional composition was calculated considering preparation as supplied by producers, considering the addition of other ingredients. This approach was used for dehydrated soups, instant beverages and cordials, mixes for cakes or desserts, as well as baby foods.

The CLAS database was further complemented with country-wide 12-month sales data. Ensuring proper data handling, we obtained sales data from two retailers covering the majority of the national market (sales data was accessible for 4 out of 5 stores included in data collection). The sales data refer to the national market and included sales of food products for the 12-month period before data collection (January 2014–December 2014). The sales data were provided in universal form, including EAN number, description of the product, the number of products sold per year, and the quantity of food (kg or L) per packaging. Matching of the foods between the databases was performed using EAN numbers. 

Initially, we surveyed 10,674 products, but for this study we only used 6619 products that had both sales data and nutritional information available. 1708 included products were labeled with NHC. 

### 2.2. Product Categorization

All data entered into the CLAS database were rechecked and each product assigned to one of the 14 parent categories and 44 predefined food categories using a classification system developed by Dunford et al. [5], which was developed as a part of the Global Food Monitoring Initiative. We used the same parent categories, but we slightly modified the categorization rules to address European market specifics. Those modifications were as follows. In the pasta category, we included pasta, noodles (pasta for soup and not Asian noodles as a prepared meal, which is a rare product in the market), and couscous. Maize was categorized depending on the product use as either a snack (popcorn) or as unprocessed cereal (flour, polenta). The parent categories and categories in our study included beverages (coffee and tea, cordials, electrolyte drinks, fruit and vegetable juices, soft drinks, and water); bread and bakery products (biscuits, bread, cakes, muffins, and pastry); cereal and cereal products (breakfast cereals, cereal bars, pasta, rice, and unprocessed cereals); confectionery (chewing gum, chocolate, sweets, and jelly); convenience foods (other, pizza, pre-prepared salads and sandwiches, ready meals, and soup); dairy (cheese, cream, desserts, ice cream and edible ices, milk, and yoghurt products), edible oils and emulsions (butter and margarine, cooking oils); eggs, fish, and fish products (canned fish and seafood, chilled fish); foods for specific dietary use (baby foods); fruit and vegetables (fruit, jam and spreads, nuts and seeds, and vegetables); meat and meat products (meat alternatives, processed meat, and derivatives); sauces and spreads (mayonnaise/dressings, sauces, and spreads); and snack foods (potato chips, crisps, and snacks) (Table S1).

### 2.3. Categorization of Claims

Products were carefully checked for labelled NHC such as nutrition claims, health claims, or front-of pack symbols. In the EU, nutrition and health claims are regulated by Regulation (EC) No 1924/2006 [11]. The list of permitted health claims was established by Commission Regulation (EU) No 432/2012 [23], which is regularly updated with newly authorized health claims. For the purpose of this study, we used regulatory definitions of nutrition and health claims as provided in Section 1. Considering the long tradition of the use of the front-of pack Protective Food symbol in Slovenia [24], foods were also checked for this symbol, which is part of a voluntarily labelling scheme operated by the Slovenian Association for Cardiovascular Health.

### 2.4. Nutrient Profiling

To determine the overall nutrition quality of foods in our dataset, two different nutrient profiling models were used—the Food Standards Australia New Zealand Nutrient Profiling Scoring Criterion (FSANZ) [15] and the WHO Regional Office for Europe (WHOE) nutrient profile model [21]. With the use of those models, foods were classified as “healthier” or “less healthy.” For the purpose of this paper, “healthier” foods are considered those permitted to carry health claims by FSANZ or those permitted for marketing to children according to WHOE. The FSANZ nutrient profiling system was chosen because it was specifically developed to determine the eligibility of a F&B to carry health claims and is already enforced in Australia and New Zealand. The WHOE nutrient profile model was chosen, because this model was specifically developed for the use in Europe, even though for other uses (restriction of food marketing). FSANZ divides foods into three categories—beverages, foods, and fats and cheese with high calcium content. WHOE sets criteria for 17 different food categories. Five categories are classified by default as “not permitted” (confectionary, sweet bakery, juices, energy drinks, and edible ices) or “permitted” (fresh and frozen meat/poultry/fish, fresh and frozen fruits/vegetable/legumes) for advertising to children. Other categories have set limits for the amounts of certain nutrients that should not be exceeded to permit marketing. The WHOE model is not applicable for use with foods intended for children under three years old; therefore, foods for specific dietary use (in our case, these were only baby foods for children under three years old) were not profiled using this model.

### 2.5. Assuring Data Quality

The accuracy of the data collection and coding was assured using a confirmation procedure. The collection of photographs was done directly in food stores. Photographs were checked for quality, and additional photographs were taken if quality was not sufficient for data extraction. After the data for a specific food were extracted into the database, they were re-checked by a second researcher. To ensure harmonized approach, all classification issues were discussed within the research team and inserted into a standard operating procedure.

### 2.6. Data Management and Statistical Analysis

Data were processed and analysed using a variety of computer programs: management of the database (Microsoft SQL Server Management Studio V13.0, Microsoft Analysis Services Client Tools 13.0, Microsoft Data Access Components (MDAC) 10.0), data collection (CLAS V1.0—Composition and Labelling Information System (Nutrition Institute, Ljubljana, Slovenia)), and data analyses (Microsoft Excel 2013 (Redmond, WA, USA) and XLSTAT 2017—Data Analysis and Statistical Solution for Microsoft Excel (Addinsoft, Paris, France)).

The nutritional quality of foods (Table 1) was assessed by comparing the mean levels of energy, total fat, saturated fat, carbohydrates, total sugars, fiber, protein, and salt of foods carrying NHC against foods that do not carry claims overall and per food category. All values are based on nutritional information, per 100 g for solid foods or per 100 mL for liquids. Mean and standard deviations (SD) were calculated. Sales-weighted energy/nutrient levels (SW) are given as exact values, and therefore, no SD is presented.

For comparison of the nutritional composition of foods and beverages with and without NHC (overall—Table 2; for food categories—Table 3) Kruskal-Wallis nonparametric test was used to determine if differences in energy and nutrient levels were statistically significant (with significance level α = 0.05 by Dunn’s procedure).

The percent agreement test was used to evaluate differences in the proportion of all F&Bs ranked as “healthier” by FSANZ and WHOE nutrient profile per food category (Table 4) and not the Cohen’s kappa because these are prescribed models to rate foods and we do not need to account for chance agreement [25]. Percent agreement was calculated by dividing the sum of products in agreement (that were ranked the same by both profiles), by sum of total products overall or per category. Agreement ranging from 90 to 100% was considered as very good agreement, 80–90% as good agreement, 70–80% as moderate agreement, 60–70% as fair agreement, 50–60% as poor agreement, and below 50% as no agreement [25].

## 3. Results and Discussion

### 3.1. Nutrition Composition of Products in the Food Supply

A study was conducted on a sample of 6619 food and drink products, categorized into 14 food categories. Categories with the highest number of products available in shops were: dairy, cereal and cereal products, beverages, bread and bakery products, and confectionery (Table 1). Beverages (soft drinks, water) and dairy (milk and yoghurt products) were the leading categories in market share in terms of sales volume, distantly followed by bread and bakery products, cereal and cereal products, and fruits and vegetables. In the following parent categories, the nutrient contents are among top three compared to other food categories for at least two nutrients that should be limited in one’s diet due to their negative impact on human health: confectionary (among top three in total sugars, saturated fat and energy), snack foods (second in energy, total fat and salt), edible oils and emulsions (on the top in energy, total fat, and saturated fat), sauces and spreads (among top three in salt and total fat), and bread and bakery products (among top three in total sugars and saturated fat).

In Table 1, we present the mean nutritional composition (for energy and selected nutrients) of available products with sales weighted means per food category, providing insights into consumers choices and preferences. In cereal and cereal products, dairy, edible oils and emulsions, eggs, and fruits and vegetables, the observed sales weighted means for energy, total fat, saturated fat, total sugar, and salt were lower than non-weighted means. This is a positive finding, indicating that in these categories products with better nutritional quality have higher market share by volume (Table 1). However, these observations should be interpreted with care because consumer food choices are dependent on many variables, and nutritional composition is usually considered as only one of those (and minor) factors. Our results indicate that, in the above-mentioned food categories, market-leading brands had lower-than-mean levels of these concerning nutrients. Conversely, sauces and spreads, confectionery, fish and fish products, foods for specific dietary use, and convenience foods were categories where consumers preferred products with lower nutrition quality, since sales weighted means for energy, total fat, saturated fat, and (for some) also total sugars were higher than mean in those categories (Table 1). These differences were considerable, up to 10 g/100 g, for some constituents. Looking at non-concerning food constituents (protein or dietary fiber), higher sales weighted contents were only observed for six categories: bread and bakery products, confectionary, convenience foods, fish and fish products, foods for specific dietary use and sauces and spreads.

### 3.2. Nutritional Composition of Products with and without Claims

Products labelled with NHC had significantly fewer calories (−197 kJ/100 g), less total fat (−4.9 g) and less saturated fat (−3.2 g), less sugar (−3.9 g), and less salt (−0.4 g) than products not labelled with NHC (Table 2). These results are comparable with results of previous Canadian [18] and European [17] studies. Products labelled with NHC also had significantly less protein (−0.8 g) content than products not carrying NHC. Conversely, carbohydrates (+0.8 g) and fiber (+0.8 g) contents were higher in foods labelled with NHC, but not significantly.

A similar pattern was also found for nutrition claims, health claims, and the FOP (front-of-pack) symbol (Table 2). However, products with the FOP symbol had notably more total fat than products with no FOP symbol, which can be attributed to the fact that a larger proportion of foods with the FOP symbol were from the edible oils and emulsions category. Products labelled with the FOP symbol had significantly less carbohydrates and less fiber. 

The sales weighted means of the nutritional composition provide additional insights about the food supply because these reflect the composition of the available foods and the popularity foods for consumers (market share). The observed sales weighted means were quite similar based on the means of all specific types of claims, with the following differences: less carbohydrates and less fiber in products with all specific types of claims and more salt (+0.1 g) for products with health claims.

In four food categories (bread and bakery products, convenience foods, eggs, and foods for specific dietary use), we observed higher salt content mean in comparison with foods not labelled with claims. With sale-weighted salt content, this trend was observed in even more food categories (Table 3). This is very concerning, considering that the salt intake in the Slovenian population exceeds the WHO recommended values by more than twofold [26]. Interestingly, similar observation was reported by Franco-Arellano and co-workers [18] for bakery products, found in Canadian food supply. On contrary in fish and fish products, we observed that in foods with NHC the salt content is considerably reduced compared to products without claims. 

In some food categories, the sales weighted means of specific concerning nutrients (or energy) for products with claims are higher than means for those nutrients. This was observed in the following categories: fruits and vegetables (energy, total fat, saturated fat, salt), foods for specific dietary use (energy, total fat, saturated fat, total sugar), fish and fish products (total fat, saturated fat, salt), meat and meat products (total fat, saturated fat, salt), cereal and cereal products (energy, total sugar), convenience foods (energy, total fat), snack foods (energy, total fats), confectionary (total sugar), bread and bakery products (salt), and beverages (salt) (Table 3). A higher market-share of products with better nutritional composition (less energy or concerning nutrients) was found in the following categories: dairy, edible oils and emulsions, fish and fish products, and sauces and spreads. 

### 3.3. Overall Nutritional Quality of Products

Nutrient profiling of the whole dataset using the FSANZ model resulted in 46% of products being considered “healthy” (Table 4). When considering sales data, the proportion of “healthy” products (volume market-share) increased to 59%. Looking at the dataset of foods labelled with NHC, 68% were determined to be “healthier” by FSANZ. The observed proportion of foods that passed the FSANZ criterion is somewhat (about 10%) higher than found in a Canadian study [18], but we should note that the results of both studies cannot be directly compared due to differences in the classification of foods and claims. However, the proportion of “healthier” products in categories with nutrition claims is similar (Table 5), with larger differences between our study and the Canadian study observed only in category of edible oils and emulsion (about 50% difference) as well as snack foods (20%). When sales data were considered, the proportion of “healthier” products (FSANZ) within the foods carrying claims increased to 75%.

The WHOE model was found considerably stricter than FSANZ; only 26% of products in the whole dataset were classified as “healthier.” However, while the FSANZ criterion was in use for restriction of health claims on foods, the WHOE was developed for restricting the advertising of foods to children. On the dataset of foods carrying claims, the proportion of WHOE “healthy” foods were 33%, and 56% when considering 12-month sales data. It seems that, in our case, products with higher market shares by volume are of better nutritional quality, thereby acknowledging that sales data are important in the assessment of the food supply. In this aspect, the category of beverages is different. In Slovenia, several educational and promotional activities have been implemented to reduce sugar consumption in drinks for several years. Also, three (unsuccessful) proposals for the introduction of a sugar/soft drinks tax have been introduced, which forced beverage producers to sign a pledge in September 2015 to limit marketing and reduce sugar content in soft drinks. This might explain our observation on the dataset of beverages carrying claims; only 19% were “healthy” per the WHOE, this percentage increased to 68% when accounting for market share, showing that consumers more often select products with lower sugar content. Notably, bottled water is included in this category, which has a large market share and is a major contributor in lower sales-weighted sugar levels when compared with non-sale weighted levels. 

Beverages are one of the best-selling categories by volume and they contain high levels of sugar. Looking at the dataset of beverages without NHC, only 37% and 22% of the market share by volume were classified as “healthy” according to FSANZ and WHOE, respectively. In contrary these percentages are much higher for beverages with NHC 69% and 68% respectively, showing strong opportunities for product reformulation with improved nutritional quality. Sales weighted means of nutritional composition provide further insight into products that are most attractive to consumers. By default, the WHOE does not permit advertising of any juices and energy drinks. For other beverages and milk drinks, sugar or sweetener must not be added. For the latter, fat should not be higher than 2.5 g/100 g. Examples of drinks in our dataset that pass the WHOE model are mainly water and water with fruit extracts. The FSANZ model is not as strict as 50% of all beverages met the requirements for »healthier« products compared with WHOE, where only 12% beverages passed as »healthier«. Within cordials, only products with sweeteners met the requirements; among soft drinks, more than 60% of those that passed the model were produced with sweeteners. Others included waters with sugar and some ingredients for taste (tea, fruit juices, and aromas); those water drinks had a mean energy of 77 kJ per 100 mL (range 32 to 115 kJ), based on total sugar mean of 4 g (range 2–5 g) per 100 mL. Some bottled mineral waters did not pass the FSANZ model due to high mineral (sodium) content. The in-depth investigation into the categories reveals information that can be used for future development of nutrient profile models. The market share of the products that pass WHOE within the coffee, cordials, and soft drinks categories is close to zero, indicating that such drinks are not popular among consumers. Water and tea showed an increase in sales weighted percent that was reflected in the as parent category from 12% to 41%. From this, we assume that water and tea are very popular beverages among Slovenian consumers.

The presented results show there is still room for improvement, especially when looking more closely at specific food categories. An example is the category of bread and bakery products, which is one of the most commonly sold food categories and has a high prevalence of health claims (21%). According WHOE, bread and bakery products are divided into three subgroups. The first is cake, sweets, and biscuits (by default non-permitted). Secondly, some of those foods in the cakes, muffins, and pastry category are rated as “healthier” according to the FSANZ model (rice cakes for example). For the third category of bread and crispy bread, the differentiating criterion is salt content: WHOE fails any bread with salt content higher than 1.2 g/100 g, whereas FSANZ passes these foods. The salt mean of those products is 1.4 g with a maximum of 1.7 g. The second differentiating factor for bread and bakery products is total fat content set by WHOE if higher than 10 g. Therefore, only few bread products (mostly dough and tortillas) do not pass WHOE standard less than 10g of total fat, but they do pass FSANZ. The market share of breads that pass WHOE as “healthier” indicates that less salty breads are more popular among consumers, but we should note that, in Slovenia, bread is also sold as non-prepacked food, and those were not included in these analyses. Bread and bakery products are an example of a food category that uses a health claim related to a single nutrient (fiber). A sub-category of breads carrying claims had significantly increased dietary fiber levels (3.5 g/100 g; Table 3). However, the total fat, saturated fat, and salt contents were also higher. Also, when bread and bakery products carrying claims were evaluated with the nutrition profiles models, only 37% and 18% pass FSANZ and WHOE criteria, respectively. Even worse results were observed for bread and bakery products not carrying claims, at 16% and 4%, respectively (Table 4). When we considered market shares, the difference between the bread and bakery products carrying and not carrying claims practically disappeared (with claims: FSANZ 42% and WHOE 20%; without claims: FSANZ 38% and WHOE 21%). A closer look at the parent category (Table S1) of bread and bakery products revealed that the least “healthy” sub-category is biscuits (with claims: FSANZ 1%, WHOE 0%; without claims: FSANZ 0%, WHOE 0%; and 20% of foods were labelled with claims), followed by cake, muffins, and pastry (with claims: FSANZ 67%, WHOE 0%; without claims: FSANZ 9%, WHOE 0%). About 70% of breads carrying claims were “healthier” according to FSANZ and 43% met the WHOE criteria. This example shows that, in some food categories, a substantial proportion of products labelled with any type of health claim do not pass any of the tested nutrient profile models. In such cases, a closer examination of the subcategories of the food parent category could reveal notable differences in the nutritional quality of foods, which is hidden in the overall proportion of parent category.

A similar trend was observed in category of beverages, where the proportion of products passing FSANZ and WHOE was much higher for foods labelled with claims (with claims: FSANZ 72%, WHOE 19%; without claims: FSANZ 38%, WHOE 9%), but our detailed look at the sub-categories demonstrated a much less optimistic situation within soft drinks (with claims: FSANZ 47%, WHOE 3%; without claims: FSANZ 12%, WHOE 0%), and a simultaneously very high prevalence of claims (30%) was observed (Table S1). This is another example that shows that studies investigating the food supply must look beyond the parent category to identify food categories that have lower nutrition quality and high use of claim. Another example confirming this finding are flavored yogurt drinks, where claims were found on 83% of foods, but among them, less than 50% and 20% of products passed FSANZ and WHOE criteria, respectively (Table S1). Considering that nutrition and health claims have an impact on consumer food choices [10], and that at least some food producers are still using those claims on foods with low nutritional quality, regulatory implementation of nutrient profiling is needed to protect consumers against misleading practices. Such an intervention could drive “healthier” food choices, providing benefits for individual and public health.

### 3.4. Comparison of Nutrient Profiling Models for Assessing the Nutritional Quality of Foods

The FSANZ and WHOE models were compared by calculating the percent of agreement between both models (Table 4). A moderate agreement (76%) was observed despite the fact that the FSANZ model operates with only three food categories, whereas WHOE is much more detailed with 20 food categories. It should be mentioned again that models are also very different in the profiling concept and targeted nutrients/constituents; for example, while FSANZ favor foods high in dietary fiber, protein and fruit/vegetables/nuts/legumes, WHOE is limiting nutrients of concern. Nevertheless, very good or good agreement were observed for many of the parent categories. Fair or poor agreement (0–70%) was observed in four categories: beverages (especially for juices and nectars as well as electrolyte drinks and coffee mixes), convenience foods (pizzas, prepared salads and sandwiches), dairy (milk, yoghurt products, and desserts), and fruit and vegetables (canned fruit, dried fruit, and nuts and fruit mixes) (Table S1). 

Of all products, 24% are rated differently in the FSANZ and WHOE models. Mostly (21%), these were rated as »healthier« by FSANZ but not by WHOE, and only 3% were rated as “healthier” by the WHOE, but not according to FSANZ.

FSANZ and WHOE differently rate breakfast cereals, as 66 pass the FSANZ but not the WHOE model, of which 59 did not pass due to the lower sugar content permitted by the WHOE (above 15 g/100 g). For the other seven breakfast cereals, the reason for the different profiling result was due to higher total fat content or salt content.

Convenience foods are rated differently primarily because of a salt content of less than 1 g is required by WHOE (for 94 products). The mean salt level in products that passed FSANZ is 1.3 g. Secondly (for 14 products), the reason for the difference was the energy limit imposed by the WHOE (941 kJ/100 g), whereas the mean energy level in those permitted by FSANZ is 1089 kJ. In the soups category, FSANZ is stricter than WHOE, mostly due to the salt content. A similar situation was observed in category of edible oils and emulsions (12 products, mostly cooking oils, passed WHOE and not FSANZ).

In the dairy category, we closely examined two subcategories: milk and yogurt products. In the milk subcategory, 71 products were permitted by FSANZ and not by WHOE. For many of them, added sugar is the differentiating factor. The total fat was differentiating factor for all whole fat milks (*n* = 26). Notably, in the WHOE, the threshold for total fat is set to 2.5 g/100 g, which is below the whole fat milk fat content (>3.5 g/100 g). Similarly, added sugar was the differentiating factor for flavored yoghurt products (*n* = 162) or total fat content for plain yogurts (*n* = 38). 

Meat and meat products is the only parent category where FSANZ is more restrictive than WHOE. Most products in our dataset were categorized by WHOE category “Processed meat and poultry, fish, and similar,” which has specific criteria for total fat (<20 g) and salt (<1.7 g). Lower FSANZ scores were mostly due to of the saturated fatty acids and salt contents. The sale-weighted data revealed that consumers prefer meat and meat products that had higher fat and salt contents.

The fruits and vegetables category is also rated differently by the two models: 242 products passed FSANZ but not WHOE (118 fruits, 94 vegetables, and the others are nuts and seeds and jams and spreads). Of the 242 fruit and vegetable products, 118 had added sugar, which was the most important factor for this difference. The other 124 products were mainly dried fruits (*n* = 65) and vegetables (*n* = 39). Dried fruit had a total sugar content higher than 10 g/100 g (on average 46 g sugar per 100 g), whereas coconut flour is high in total fat. 

A strength of this study is in the large dataset that reflects the situation in the national food supply. Access to 12-month sales data enabled us to use the sale-weighting approach, providing deeper insights into the food supply. This is an innovative approach in the area of public health research because sales data are usually expensive and not available to research organizations. In our case, sales data were provided free of charge by retailers under the condition that the data would only be used for research and that sales data of any individual food product would not be publicly disclosed. 

A major limitation for the interpretation of the results of this study is the fact that different food categorization systems have been used in different studies. To ensure comparability of the results with future studies, we used the categorization system developed by the Global Food Monitoring Group [5]. The representativeness of the sample is also an important issue in studies that investigate the food supply. Sampling for this study was performed in grocery stores in an urban area, but we should note that only retailers with nation-wide networks of shops were selected. The retailers that provided sales data covered over 50% of the national market in terms of volume. Another limitation of the study is that the used approach is only applicable for the assessment of prepacked foods. We should note that in some food categories (particularly in breads and bakery foods, fruits and vegetables, and fresh meat), non-prepacked foods have considerable market-shares.

## 4. Conclusions

The reformulation of food products available in the food supply is recognized as one of the key factors that could improve unhealthy diets and reduce the public health burden of noncommunicable diseases. Therefore, the food supply needs to be monitored from a variety of aspects. We should focus on specific constituents of concern such as sodium [7], sugar [19], and trans fatty acids [27], which were investigated in our previous studies. The overall nutritional quality of the foods also needs to be considered. In this study, we investigated the nutritional quality of foods that were or were not labelled with nutrition and health claims and applied the sale-weighing approach to gain insights into consumer preferences when buying such products. Application of market-share data is important because this enables the differentiation between niche and market-leading products. The latter are those that have a greater effect on public health and need to be monitored even more carefully. Our study showed that foods labelled with health claims, on average, had somewhat fewer calories, and less total fat, saturated fat, sugar, and salt, in comparison with products not labelled with claims. However, considerable proportions of such foods were found to have inappropriate nutritional composition to be promoted by health claims. About a third of foods labelled with claims did not pass FSANZ criterion, and even more concerning results were obtained when applying the WHOE model for nutrition profiling.

Despite our observations that, in some food categories, the market leading products had a better than average nutritional profile, the results of this study highlight the need for stronger regulation of the use of nutrition and health claims. It is not yet clear how this will affect the food supply and if such an intervention would be reflected in measurable differences in health outcomes, but it is clear that we should build on available nutrient profiling knowledge to improve nutrition quality of foods carrying health claims, allowing consumers to make healthy choices easily.

## Figures and Tables

**Table 1:** Overview of foods in the market, their mean nutritional values with standard deviation and sales weighted mean per food category. *n* denotes number of products; SW denotes sales weighted mean.

		Food Category													
		Beverages	Bread and Bakery Products	Cereal and Cereal Products	Confectionery	Convenience Foods	Dairy	Edible Oils and Emulsions	Eggs	Fish and Fish Products	Foods for Spec. Dietary Use	Fruit and Vegetables	Meat and Meat Products	Sauces and Spreads	Snackfoods
	*n*	796	794	803	696	406	1133	209	18	166	121	674	300	312	191
Nutrient (per 100 g or per 100 mL)															
Energy (kJ)	Mean	141	1733	1504	1911	531	643	3210	616	877	322	827	1095	1150	2043
	SD	73	375	249	512	417	427	666	41	408	258	809	502	936	234
	SW Mean	93	1591	1502	2029	578	375	3191	613	1123	380	540	1022	1643	2051
Total fat (g)	Mean	0.2	15.8	4.9	21.5	5.2	9.4	86.5	10.6	14.6	1.4	9.4	20.8	21.7	23.9
	SD	0.5	9.6	5.6	15.8	5.0	10.2	18.3	1.0	11.5	1.7	18.5	13.6	23.9	9.0
	SW Mean	0.0	12.5	3.2	24.6	5.8	5.3	86.1	10.5	22.1	2.5	5.3	20.3	31.6	24.4
Saturated fat (g)	Mean	0.1	7.4	1.8	11.7	1.7	6.2	23.3	2.9	2.5	0.6	2.2	6.5	4.0	6.7
	SD	0.3	5.9	2.9	9.2	2.0	7.1	20.5	0.3	1.7	0.9	7.1	5.3	5.0	4.4
	SW Mean	0.0	5.9	1.1	13.1	1.8	3.4	19.4	2.9	3.6	1.5	1.1	6.1	5.6	6.4
Carbohydrates (g)	Mean	7.6	59.1	66.1	63.1	13.5	11.4	0.3	1.0	2.9	13.8	22.6	2.4	15.6	57.8
	SD	4.1	12.4	12.8	17.7	12.7	8.9	0.8	0.3	5.3	11.7	23.1	3.0	17.8	8.8
	SW Mean	5.3	57.4	70.1	61.4	14.8	6.6	0.2	1.0	2.6	13.7	14.3	1.4	23.7	57.2
Total sugars (g)	Mean	7.0	20.9	8.2	46.5	1.9	9.4	0.2	0.8	0.8	7.9	16.1	0.6	11.8	3.2
	SD	3.9	16.1	10.8	21.5	2.6	7.4	0.7	0.3	1.4	4.0	21.2	0.7	17.3	5.1
	SW Mean	5.0	18.5	5.3	50.4	1.8	6.2	0.2	0.8	1.1	9.3	7.8	0.4	21.6	2.5
Fibre (g)	Mean	0.2	3.6	4.9	2.6	1.6	0.3	0.0	0.0	0.5	0.8	4.5	0.9	1.9	4.1
	SD	0.5	2.8	4.1	3.1	1.7	0.5	0.2	0.0	0.7	0.8	4.9	1.2	2.0	1.9
	SW Mean	0.0	3.2	3.6	2.4	1.8	0.1	0.0	0.0	0.6	1.5	4.0	0.8	2.0	3.9
Protein (g)	Mean	0.2	7.3	10.2	4.9	5.7	5.8	0.2	12.2	16.8	1.7	4.9	16.5	4.2	8.3
	SD	0.4	2.9	3.5	3.7	5.6	6.6	0.6	0.2	6.4	1.9	7.4	7.9	4.8	3.5
	SW Mean	0.1	7.5	9.9	5.3	5.8	3.9	0.2	12.2	15.4	3.0	4.5	14.3	3.3	8.3
Salt (g)	Mean	0.0	0.8	0.4	0.2	1.0	0.4	0.2	0.4	1.5	0.1	0.6	2.0	2.1	2.0
	SD	0.1	0.7	0.6	0.2	0.4	0.6	0.4	0.0	2.0	0.1	1.1	1.2	3.1	0.8
	SW Mean	0.1	0.9	0.2	0.2	0.9	0.2	0.1	0.4	1.1	0.1	0.6	2.1	1.1	2.0

**Table 2:** Comparison of the nutritional composition of foods and beverages with and without certain type of claims (*n* = 6619).

Nutrient (per 100 g or per 100 mL)	Presence of Claim	Nutrition claim (*n*_c_ = 1602; *n*_nc_ = 5017)				Health Claim (*n*_c_ = 462; *n*_nc_ = 6157)				FOP Symbol (*n*_c_ = 104; *n*_nc_ = 6515)				NHC (*n*_c_ = 1708; *n*_nc_ = 4911)			
		A	SD	*p*	SWA	A	SD	*p*	SWA	A	SD	*p*	SWA	A	SD	*p*	SWA
Energy (kJ)	Claim	973	862	<0.0001 *	316	1076	926	0.026 *	288	1067	1223	0.018 *	577	983	862	<0.0001 *	320
	No claim	1179	841		639	1133	845		548	1130	844		531	1180	841		639
Total fat (g)	Claim	9.7	20.6	<0.0001 *	4.4	12.2	23.0	<0.0001 *	4.3	17.0	35.4	0.024 *	9.4	9.8	20.4	<0.0001 *	4.4
	No claim	14.6	18.7		8.1	13.5	19.0		7.0	13.4	18.9		6.8	14.7	18.7		8.1
Saturated fat (g)	Claim	2.7	6.0	<0.0001 *	1.2	3.7	8.2	<0.0001 *	1.0	2.6	4.6	0.1007	1.7	2.8	6.5	<0.0001 *	1.2
	No claim	6.0	8.6		3.3	5.3	8.2		2.7	5.3	8.2		2.6	6.1	8.5		3.3
Carbohydrates (g)	Claim	30.8	30.3	0.791	6.8	31.4	30.2	0.6859	6.1	18.6	23.4	<0.0001 *	8.8	31.2	30.4	0.783	7.0
	No claim	30.5	27.8		16.3	30.5	28.3		13.6	30.8	28.5		13.2	30.4	27.7		16.2
Total sugars (g)	Claim	10.1	14.7	<0.0001 *	4.0	9.8	13.8	<0.0001 *	2.5	6.2	5.5	0.044 *	6.6	10.6	15.3	0.0001 *	4.0
	No claim	14.5	19.0		8.4	13.7	18.4		7.3	13.6	18.3		7.0	14.5	18.9		8.4
Fibre (g)	Claim	2.8	4.5	0.188	0.4	3.1	4.6	0.5143	0.5	2.2	3.5	0.0003 *	0.6	2.8	4.4	0.257	0.4
	No claim	2.0	2.7		0.8	2.2	3.1		0.7	2.2	3.2		0.7	2.0	2.7		0.8
Protein (g)	Claim	5.5	6.2	<0.0001 *	2.0	6.0	6.8	0.005 *	1.5	6.5	5.9	0.583	4.8	5.6	6.3	<0.0001 *	2.1
	No claim	6.4	6.4		3.4	6.2	6.3		3.0	6.2	6.4		2.9	6.4	6.4		3.4
Salt (g)	Claim	0.3	0.6	<0.0001 *	0.2	0.4	0.7	<0.0001 *	0.3	0.3	0.6	<0.0001 *	0.2	0.4	0.6	<0.0001 *	0.2
	No claim	0.7	1.3		0.3	0.7	1.2		0.2	0.7	1.2		0.3	0.7	1.3		0.3

Note: *n*_c_, number of products with health-related claim; *n*_nc_, number of products without health-related claim; FOP, front-of-pack; NHC, nutrition and health claim, A, mean; SD, standard deviation; SWA, sales weighted mean; *, statistically significant difference *p* < 0.05; All values are based on nutritional information per 100 g or per 100 mL. NHC states for any nutrition or health claims as defined within the EU Regulation 1924/2006 on nutrition and health claims [11].

**Table 3:** Comparison of the nutritional composition of foods and beverages with (*n* = 1708) and without (*n* = 4911) claims by food categories mean and sales weighted mean (SW M.).

Nutrition Information (per 100 g or per 100 mL)		Presence of NHC	Beverages	Bread and Bakery Products	Cereal and Cereal Products	Confectionery	Convenience Foods	Dairy	Edible Oils and Emulsions	Eggs	Fish and Fish Products	Foods for Specific Dietary Use	Fruit and Vegetables	Meat and Meat Products	Sauces and Spreads	Snackfoods
Energy (kJ)	Mean	Claim	117 *	1667 *	1572 *	1204 *	864 *	341 *	3140	615	818	339 *	950 *	673 *	1430	1986
		No Claim	155 *	1750 *	1458 *	2064 *	519 *	764*	3260	617	905	140 *	799 *	1177 *	1121	2051
	SW M.	Claim	35	1561	1573	1167	893	266	3072	639	816	383	963	671	1256	2019
		No Claim	132	1595	1482	2108	571	440	3262	611	1248	207	509	1,075	1667	2054
Total fat (g)	Mean	Claim	0.1 *	13.0 *	5.7 *	4.0 *	9.4 *	3.1 *	84.7	10.4	13.5	1.5 *	8.3	8.7 *	31.4 *	22.2
		No Claim	0.2 *	16.5 *	4.3 *	25.2 *	5.1 *	11.9 *	87.8	10.6	15.2	0.3 *	9.7	23.1 *	20.7 *	24.1
	SW M.	Claim	0.0	10.8	4.7	2.4	10.0	2.6	82.9	11.1	14.4	2.5	12.4	10.1	30.1	23.8
		No Claim	0.1	12.8	2.8	26.6	5.7	6.9	88.0	10.5	25.2	0.3	4.8	21.8	31.7	24.5
Saturated fat (g)	Mean	Claim	0.0 *	5.3 *	1.9 *	2.1 *	2.0	1.9 *	16.8 *	2.7	2.7	0.7	3.3	2.3 *	5.0	4.1 *
		No Claim	0.2 *	7.9 *	1.8 *	13.7 *	1.7	7.9 *	27.9 *	2.9	2.4	0.1	1.9	7.3 *	3.9	7.1 *
	SW M.	Claim	0.0	4.1	1.6	1.3	2.0	1.7	12.5	3.0	3.0	1.5	5.9	3.2	3.4	2.8
		No Claim	0.0	6.2	1.0	14.2	1.7	4.5	23.6	2.9	3.8	0.1	0.8	6.6	5.7	6.8
Carbohydrates (g)	Mean	Claim	6.4 *	59.3	66.7 *	76.4 *	15.0	8.8 *	0.2 *	0.7	1.8	14.4	33.8 *	2.7	10.5	55.5
		No Claim	8.3 *	59.1	65.7 *	60.2 *	13.5	12.4 *	0.4 *	1.1	3.4	7.0	20.1 *	2.3	16.1	58.1
	SW M.	Claim	1.9	55.7	69.8	76.4	15.3	6.3	0.2	1.2	3.0	13.8	22.9	1.7	6.8	58.0
		No Claim	7.6	57.7	70.1	60.1	14.8	6.8	0.3	1.0	2.5	10.6	13.7	1.4	24.7	57.1
Total sugars (g)	Mean	Claim	5.8 *	11.6 *	12.8 *	28.1 *	2.0	7.4 *	0.2 *	0.6	1.1	8.2	25.4 *	0.7	6.5	4.1
		No Claim	7.6 *	23.4 *	5.1 *	50.5 *	1.9	10.3 *	0.2 *	0.8	0.7	5.0	14.0 *	0.6	12.3	3.1
	SW M.	Claim	1.8	11.4	14.3	30.9	1.4	5.9	0.2	1.0	2.4	9.3	15.0	0.7	4.9	4.0
		No Claim	7.2	19.5	2.9	52.2	1.9	6.3	0.2	0.8	0.5	7.6	7.3	0.4	22.7	2.4
Fiber (g)	Mean	Claim	0.2	6.4 *	7.2 *	1.3 *	2.6	0.4	0.0	0.0	0.7 *	0.9 *	5.4	1.4 *	3.2	4.9
		No Claim	0.1	2.9 *	3.4 *	2.9 *	1.5	0.2	0.0	0.0	0.4 *	0.4 *	4.3	0.9 *	1.8	4.0
	SW M.	Claim	0.0	6.9	6.2	0.8	2.1	0.1	0.0	0.0	1.2	1.5	6.0	0.8	1.7	4.4
		No Claim	0.1	2.7	2.9	2.5	1.8	0.0	0.0	0.0	0.4	0.6	3.9	0.8	2.0	3.9
Protein (g)	Mean	Claim	0.2 *	8.5 *	10.4	1.9 *	13.9 *	4.3 *	0.2 *	12.2	17.0	1.8 *	4.4 *	16.8 *	6.5	9.5
		No Claim	0.3 *	6.9 *	10.1	5.5 *	5.4 *	6.5 *	0.2 *	12.2	16.6	0.5 *	5.0 *	16.5 *	4.0	8.2
	SW M.	Claim	0.0	9.5	10.1	0.8	14.6	3.7	0.1	12.1	13.6	3.0	6.0	15.7	1.8	6.9
		No Claim	0.1	7.3	9.8	5.7	5.6	4.0	0.2	12.2	16.1	0.6	4.4	14.1	3.4	8.5
Salt (g)	Mean	Claim	0.0 *	0.9 *	0.3 *	0.1 *	1.4 *	0.2 *	0.2	0.4	1.0 *	0.1 *	0.3 *	1.5 *	1.2	1.7 *
		No Claim	0.0 *	0.8 *	0.4 *	0.2 *	1.0 *	0.5*	0.1	0.3	1.8 *	0.0 *	0.7 *	2.1 *	2.2	2.0 *
	SW M.	Claim	0.1	1.0	0.3	0.0	1.2	0.1	0.1	0.4	0.9	0.1	0.6	1.8	1.4	1.8
		No Claim	0.0	0.8	0.2	0.2	0.9	0.2	0.1	0.4	1.1	0.0	0.6	2.2	1.1	2.0

Note: * denotes statistically significant difference (*p* < 0.05). All values are based on nutritional information, per 100 g or per 100 mL for products as consumed. Claim states for any nutrition or health claims as defined within the EU Regulation 1924/2006 on nutrition and health claims [11].

**Table 4:** Proportion of foods eligible by two nutrition profile models—Australia/New Zealand (FSANZ) and European World Health Organization Regional office for Europe model (WHOE); per category (all products, products with claims, and products without claims) and the difference with sales weighted data.

Food Categories	All Products						Products with NHC					Products with no NHC				
	*n*	FSANZ		WHOE		PA	*n*	FSANZ		WHOE		*n*	FSANZ		WHOE	
		%	SW %	%	SW %			%	SW %	%	SW %		%	SW %	%	SW %
Beverages	796	50%	50%	12%	41%	63%	281	72%	69%	19%	68%	515	38%	37%	9%	22%
Bread and bakery products	794	20%	39%	7%	21%	86%	166	37%	42%	18%	20%	628	16%	38%	4%	21%
Cereal and cereal products	803	77%	85%	65%	80%	87%	322	73%	67%	53%	54%	481	79%	90%	72%	87%
Confectionery	696	9%	5%	0%	0%	91%	124	45%	48%	0%	0%	572	1%	1%	0%	0%
Convenience foods	406	61%	71%	49%	53%	58%	14	79%	86%	21%	31%	392	61%	71%	50%	54%
Dairy	1133	51%	73%	19%	26%	60%	323	76%	86%	42%	39%	810	41%	64%	10%	18%
Edible oils and emulsions	209	67%	76%	73%	77%	94%	86	80%	95%	88%	98%	123	59%	64%	62%	65%
Eggs	18	100%	100%	100%	100%	100%	2	100%	100%	100%	100%	16	100%	100%	100%	100%
Fish and fish products	166	57%	43%	65%	52%	90%	53	64%	79%	79%	95%	113	54%	29%	58%	34%
Foods for specific dietary use	121	99%	100%	NA	NA	NA	111	99%	100%	NA	NA	10	100%	100%	NA	NA
Fruit and vegetables	674	73%	92%	38%	53%	64%	125	73%	85%	26%	29%	549	73%	92%	40%	55%
Meat and meat products	300	21%	10%	26%	24%	87%	49	59%	38%	55%	29%	251	13%	5%	21%	23%
Sauces and spreads	312	22%	18%	2%	1%	80%	29	38%	25%	3%	2%	283	20%	18%	2%	1%
Snackfoods	191	5%	1%	1%	1%	96%	23	9%	0%	0%	0%	168	4%	1%	1%	1%
All products	6619	46%	59%	26%	37%	76%	1708	68%	75%	33%	56%	4911	39%	51%	23%	27%

Note: PA, percent of agreement. Claim states for any nutrition or health claims as defined within the EU Regulation 1924/2006 on nutrition and health claims [11].

**Table 5:** Proportion of foods eligible by two nutrition profile models—Australia/New Zealand (FSANZ) and European World Health Organization Regional office for Europe model (WHOE); per category (products with nutrition claims and products with health claims) and the difference with sales weighted data.

Food Categories	Products with Nutrition Claims					Products with Health Claims				
	*n*	FSANZ		WHOE		*n*n	FSANZ		WHOE	
		%	SW %	%	SW %			%	SW %	%	SW %
Beverages	259	70%	68%	18%	68%	79	80%	31%	27%	93%
Bread and bakery products	155	39%	44%	19%	21%	45	22%	31%	11%	7%
Cereal and cereal products	304	75%	67%	55%	53%	98	55%	51%	40%	42%
Confectionery	103	50%	58%	0%	0%	49	59%	61%	0%	0%
Convenience foods	7	86%	87%	14%	0%	7	71%	85%	29%	45%
Dairy	318	76%	86%	42%	39%	62	66%	67%	37%	38%
Edible oils and emulsions	83	82%	95%	90%	98%	34	74%	90%	85%	98%
Eggs	2	100%	100%	100%	100%	1	100%	100%	100%	100%
Fish and fish products	51	63%	78%	78%	95%	9	56%	67%	78%	80%
Foods for specific dietary use	109	99%	100%	0%	0%	29	97%	99%	0%	0%
Fruit and vegetables	115	71%	80%	26%	32%	24	88%	100%	42%	29%
Meat and meat products	49	59%	38%	55%	29%	18	56%	49%	17%	10%
Sauces and spreads	26	38%	24%	4%	2%	4	25%	24%	0%	0%
Snackfoods	21	10%	0%	0%	0%	3	0%	0%	0%	0%
All products	1602	69%	75%	34%	56%	462	63%	41%	30%	79%

Note: Nutrition and health claims as defined within the EU Regulation 1924/2006 on nutrition and health claims [11].

## Supplementary Materials

The following are available online at http://www.mdpi.com/2072-6643/11/2/287/s1, Table S1: Proportion of prepacked foods per food category based Dunford categorization that pass FSANZ and WHOE nutrient profile labelled with health-related claims.
